# Global Positioning System-Based Stimulation for Robo-Pigeons in Open Space

**DOI:** 10.3389/fnbot.2017.00040

**Published:** 2017-08-14

**Authors:** Junqing Yang, Ruituo Huai, Hui Wang, Wenyuan Li, Zhigong Wang, Meie Sui, Xuecheng Su

**Affiliations:** ^1^Institute of RF and OE-ICs, Southeast University, Nanjing, China; ^2^College of Electrical Engineering and Automation, Shandong University of Science and Technology, Qingdao, China

**Keywords:** brain–computer interface, robo-pigeon, bio-robot, flight control, stimulator

## Abstract

An evaluation method is described that will enable researchers to study fight control characteristics of robo-pigeons in fully open space. It is not limited by the experimental environment and overcomes environmental interference with flight control in small experimental spaces using a compact system. The system consists of two components: a global positioning system (GPS)-based stimulator with dimensions of 38 mm × 26 mm × 8 mm and a weight of 18 g that can easily be carried by a pigeon as a backpack and a PC-based program developed in Virtual C++. The GPS-based stimulator generates variable stimulation and automatically records the GPS data and stimulus parameters. The PC-based program analyzes the recorded data and displays the flight trajectory of the tested robo-pigeon on a digital map. This method enables quick and clear evaluation of the flight control characteristics of a robo-pigeon in open space based on its visual trajectory, as well as further optimization of the microelectric stimulation parameters to improve the design of robo-pigeons. The functional effectiveness of the method was investigated and verified by performing flight control experiments using a robo-pigeon in open space.

## Introduction

Some animals have amazing senses of smell that enable them to detect narcotics, explosives, and pipeline leaks (Britt et al., 2008). Engineers have not developed any device with odor detection capabilities comparable to those of canines. Furthermore, some animals can traverse a variety of terrain types more efficiently than electromechanical robots or humans (Grinke et al., 2015); for instance, engineers have not developed a micro air vehicle with flight abilities comparable to those of pigeons. Animals could be employed to conduct search and rescue missions more efficiently than electromechanical robots if they could be controlled. Therefore, increasing interest is developing in the prospect of controlling animals and utilizing them as new kinds of robots.

Special kinds of bionic robots called bio-robots have been developed based on brain–computer interfaces (BCIs), which involve direct communication between the brain and an external device. The first BCI-based robo-rat was developed according to a “virtual reward” behavior model in 2002 (Talwar et al., 2002). Micro-electrodes were implanted into three regions in a rat’s brain: the medial forebrain bundle (MFB) and the whisker representations in the left and right somatosensory cortices (SIs). The MFB was stimulated to generate intense excitement as a virtual reward, while the left and right SIs were stimulated to generate “virtual touches” in the corresponding directions. With stimulation of both the MFB and SIs, the robo-rat could be trained to perform certain behaviors, such as “walk forward,” “turn left,” and “turn right” in certain circumstances (Xu et al., 2004). These BCI-based bio-robots were developed successfully, and proof-of-concept tests were conducted in which motion along a complicated route was performed by remote control, even in 3-D terrain, by utilizing a telemetry system for brain microstimulation. BCI-based bio-robots are different from electromechanical robots as they are controlled by electrical stimulation of specific regions within the brains of the employed animals. In addition, bio-robots incorporate animals’ visual, audio, and tactile sensory capabilities, which increase their intelligence without requiring any extraneous attachments. Bio-robots’ movements are not dependent on motors, which are necessary in electromechanical robots and have high energy consumptions. Consequently, bio-robots are not limited by energy shortage capabilities when traveling over long distances and are more skilled than electromechanical robots when conducting complex missions. As bio-robots are superior to electromechanical robots in many potential applications, researchers have been investigating different types of bio-robots, such as rats (Feng et al., 2007; Huai et al., 2009; Pi et al., 2010; Zhang et al., 2012; Su et al., 2014; Zheng et al., 2015; Yu et al., 2016), geckos (Guo et al., 2009), sharks (Gomes et al., 2006), goldfishes (Kobayashi et al., 2009), carps (Peng et al., 2011), cockroaches (Holzer and Shimoyama, 1997), pigeons (Su et al., 2012), beetles (Hirotaka et al., 2008), and honeybees (Bao et al., 2011).

We developed the first BCI-based robo-pigeon using a new “virtual fear” behavior model in 2007 (Xinhua, 2007). In this type of robo-pigeon, microelectrodes are implanted into three motion-related nuclei in the brain: the left and right dorsalis intermedius ventralis anterior (DIVA) nuclei and the periaqueductal gray (PAG) region. The robo-pigeon can then be controlled *via* neural reactions to functional electrical stimulation. In this method, charge is transferred into the three motion-related regions in the brain of the robo-pigeon, externally exciting the membrane potentials of the neurons and inducing the neurons to fire in response. The efficacy of a robo-pigeon closely depends on the amount of charge transferred to the three motion-related neural tissues. The transferred charge is determined by several factors, including the frequency, number, and duration of the stimulation pulses, as well as the locations and surface coating of the stimulating electrodes. It is impractical to maintain the same precise electrode positioning in the brains of different pigeons (i.e., pigeons of different species, weights, or ages) during surgical implantation. Electrode positioning inaccuracy makes it essential to vary and optimize the stimulus settings in every stimulation channel for each robo-pigeon.

We previously reported on the remote control of robo-pigeons using a conventional neural stimulator with an RF transceiver (Yang et al., 2015). Forward motion and turns to the left and right were achieved by stimulating the PAG region and the left and right DIVA nuclei, respectively, by utilizing a telemetry system for brain microstimulation in laboratory environments, i.e., small enclosed spaces, typically experimental chambers or workshops, which are completely different from the open spaces in which robo-pigeons would actually be employed, limit their flight, and interfere with optimization of the stimulus settings. In addition, robo-pigeons would be out of the sight of their handlers in open space, and the telemetry system previously employed in laboratory environments is useless in such situations. These problems will be solved by the new system and method described in this paper, which can be utilized for flight control and optimization of the stimulus settings of robo-pigeons in open space.

## Materials and Methods

### Overview

We describe the design of the system in the context of robo-pigeon fight control experiments in this section. The system consists of two separate components: an integrated global positioning system (GPS)-based microstimulator and a customized C++ program. The former is mounted on the back of a robo-pigeon and connected to the electrodes implanted in its brain and is responsible for generating micro-electrical stimulation and recording the experimental data during the flight control test. The latter is run on a PC or laptop and is in charge of analyzing the experimental data and displaying the results.

### GPS-Based Microstimulator

The GPS-based microstimulator in the proposed system is primarily composed of a microprocessor (ATmega8L, Atmel Inc.), a trans-flash (TF) card module, and a micro GPS module (SR-92) with a built-in patch antenna (ProGin Technology Inc.). All of the components are assembled on a printed circuit board and powered by a 3.7 V polymer battery.

**Figure 1:**
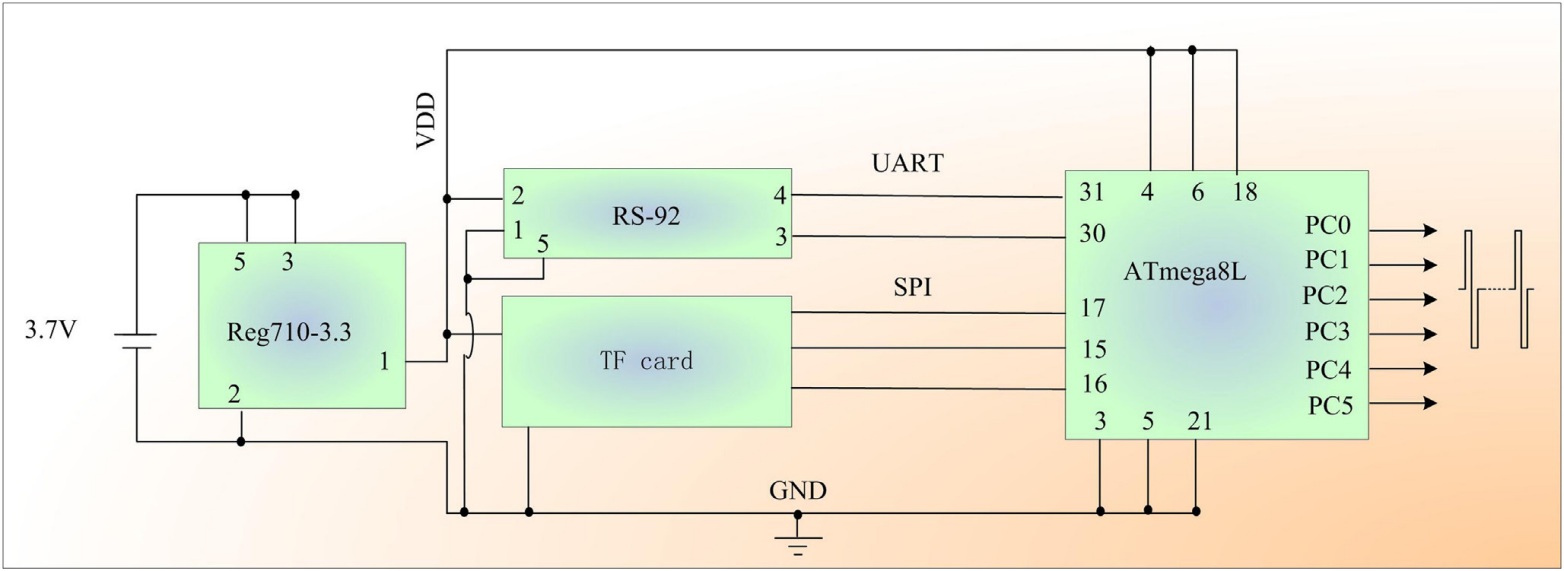
Schematic diagram of the global positioning system-based robo-pigeon microstimulator.

Figure 1 illustrates the circuitry of the GPS-based microstimulator in detail. Reg710-3.3 is a 3.3 V regulator. The programmable microprocessor (ATmega8L) has 23 digital I/O pins: two of these pins are employed to communicate with the GPS module as a serial port, and three pins are used to operate the TF card as a serial peripheral interface (SPI) port. Six pins are retained to generate a biphasic pulse as three separate output channels. In each output channel, two pins (PC0 and PC1, PC2 and PC3, or PC4 and PC5) are used to stimulate one of the three motion-related brain regions with constant voltage biphasic stimulus pulses. The GPS-based microstimulator also records the experimental data, including the location and velocity of the bio-robot and the corresponding stimulation parameters (pulse number, duration, and frequency) during its flight.

One program runs on the microprocessor onboard the robo-pigeon and generates gradient stimulation in every output channel of the GPS-based microstimulator. After the flight control test has begun, each channel is initiated with the same stimulation parameters: pulse number = 2, pulse duration = 0.2 ms, and pulse frequency = 80 Hz. Then, the corresponding pulse trains are applied alternately to stimulate the three motion-related brain regions. Simultaneously, the GPS data and stimulation parameters are recorded on the TF card. During the robo-pigeon flight control test, the stimulus is delivered independently to DIVA or PAG according to different circumstances of flight test or take-off test. The stimulus parameters are increased gradually and alternately according to the following rules: the pulse duration, number, and frequency are increased in increments of 1 (unit: 0.1 ms), 5, and 10, respectively. One parameter is changed in each stimulation cycle until each parameter reaches its maximum value (pulse number = 20, pulse duration = 0.8 ms, pulse frequency = 120 Hz.).

The microprocessor receives the GPS data and extracts the latitude, longitude, and speed from the GPRMC [one of many sentences in the National Marine Electronics Association (NMEA) standard for GPS receiver, for all sentences start with GP, RMC-recommended minimum data for GPS]-formatted GPS data stream. These data and the corresponding stimulation parameters are written into a file allocation table file on the TF card as a record with the format shown in Table 1. Each record is composed of a pre-header (PH), GPRMC-formatted GPS data, a command, and stimulation parameters. For example, in “@3559.8758, N, 12006.8668, E, 20.91, L, 10, 4, 80,” “@” is a header identifying the start of the record, the location of the object is 35°59.8758′ N and 120°6.8668′ E, the speed of the object is 20.91 knots (1 knot = 1.852 km/h), “L” indicates stimulation of the left DIVA (L: stimulation of left DIVA, R: stimulation of right DIVA, T: stimulation of PAG, I: idle), and the stimulation parameters are: pulse number = 10, pulse duration = 0.4 ms, and pulse frequency = 80 Hz. Each update of the GPS data triggers a new recording; therefore, all of the experimental data are recorded one by one on the TF card during the flight of the robo-pigeon in open space.

**Table 1:** Formatted data recording.

Pre-header	GPRMC-formatted global positioning system data	Command	Stimulation parameters		
PH	Latitude, N/S, longitude, E/W, speed	L/R/T/I	Pulse number	Pulse duration	Pulse frequency
@	3559.8758, N, 12006.8668, E, 20.91	L	10	4	80

### Data processing

**Figure 2:**
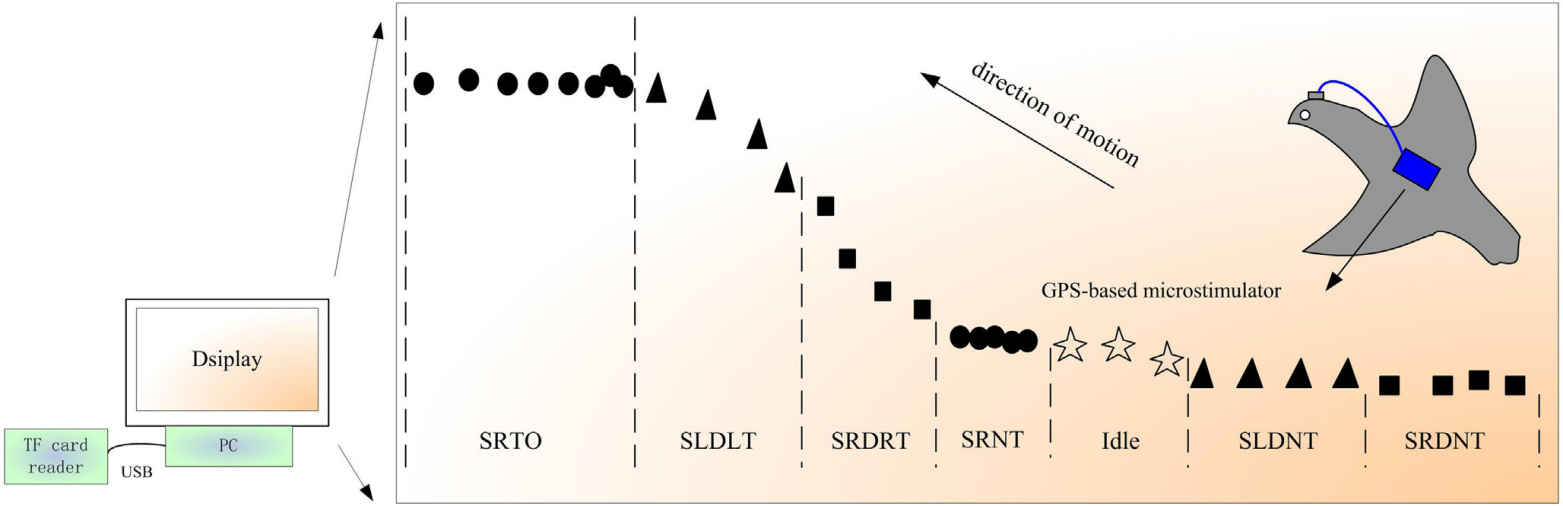
Schematic diagram of the data processing system and example results.

The proposed data processing system includes a TF card reader and a PC-based program developed in Visual C++. The program processes the experimental data that are recorded on the TF card and displays the experimental results, as shown in Figure 2. The results are presented in the form of the 2-D trajectory of the investigated robo-pigeon that is drawn automatically by the program on a digital map based on the experimental data. Each trajectory is composed of numerous dots with four different shapes. Each dot corresponds to a data record and a position of the bio-robot during its flight. The shapes represent the following command types: ▲ = L command, ■ = R command, ● = T command, and ☆ = idle, i.e., without a command.

The trajectory in Figure 2 demonstrates how the control characteristics of the robo-pigeon can be evaluated. Each segment of the trajectory can be categorized as corresponding to effective control or ineffective control. The effective control situations include stimulating the right DIVA nucleus and inducing a right turn (SRDRT), stimulating the left DIVA nucleus and inducing a left turn (SLDLT), and stimulating the PAG region and inducing take-off (SRTO), while the ineffective control situations include stimulating the right DIVA nucleus but not inducing a turn (SRDNT), stimulating the left DIVA nucleus but not inducing a turn (SLDNT), and stimulating the PAG region but not inducing take-off (SRNT). For effective control, the stimulus parameter settings can be determined and optimized for every stimulation channel and the corresponding stimulation parameters recorded. If ineffective control is observed, further investigation should be conducted on the locations and surface coating of the stimulating electrodes and robo-pigeon design optimization strategies should be identified.

## Results

**Figure 3:**
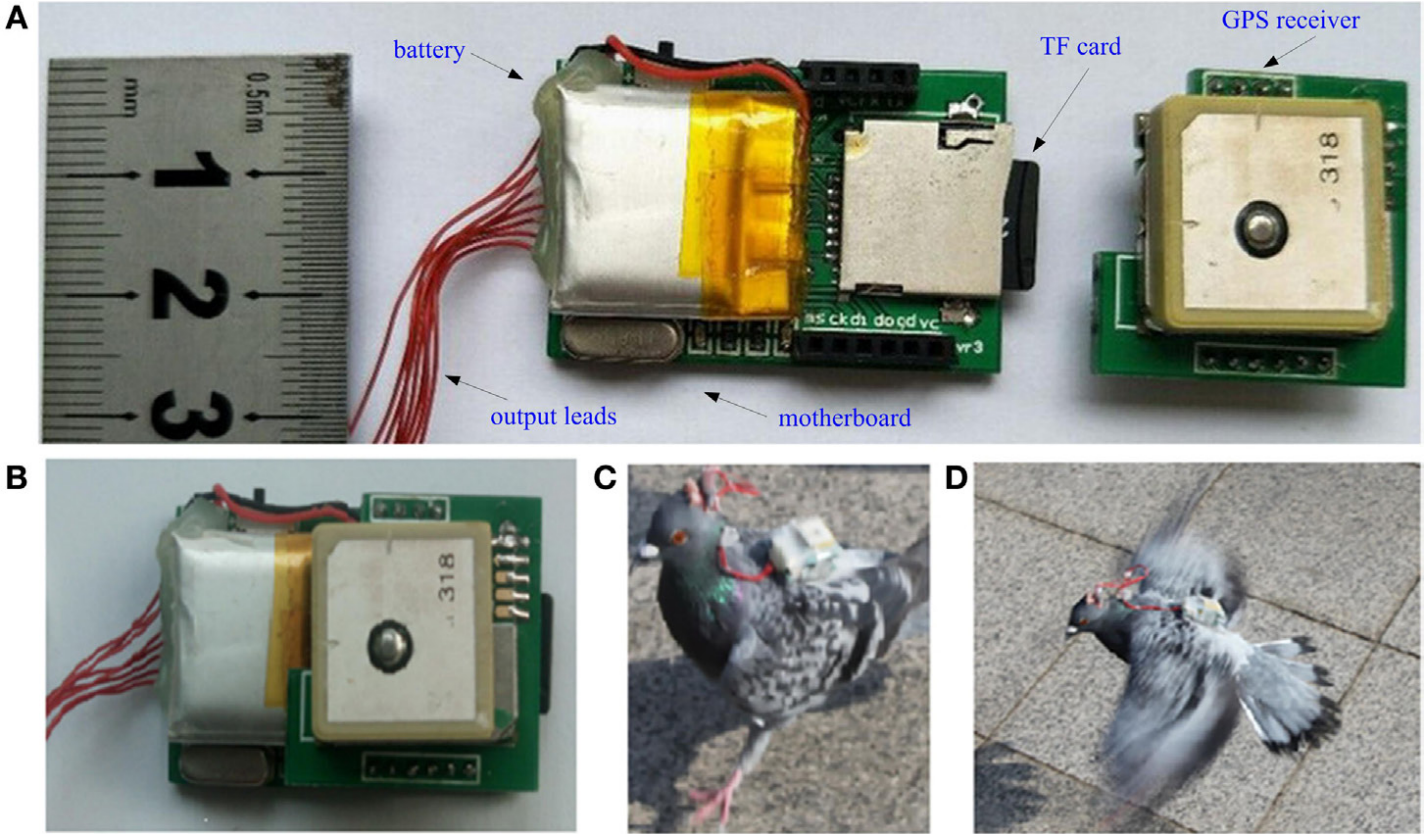
Photographs of the global positioning system (GPS)-based microstimulator and robo-pigeon test in open space. **(A)** Unassembled unit modules, **(B)** assembled GPS-based microstimulator, **(C)** landing, and **(D)** flying robo-pigeon in test.

We manufactured the modules shown in Figure 3A: a micro-GPS module, output leads, a TF card, and a motherboard. All of the other units were mounted on the motherboard through slots to assemble the GPS-based microstimulator depicted in Figure 3B. The GPS-based microstimulator was powered by a rechargeable 3.7 V, 240 mAh polymer battery attached to the back side of the motherboard and electrically connected to the motherboard when used by turning on the microswitch. The battery of the system lasts about 2.3 h, which is enough for conducting a complete test of robo-pigeon, compared with an average of slightly more than 1 h needed for each test. The GPS-based microstimulator was mounted on the back of a robo-pigeon as a backpack, and the terminals of the six output leads were connected to the stimulating electrodes located in the three brain regions (the left and right DIVA nuclei and the PAG region). The backpack measured 38 mm × 26 mm × 8 mm, weighed 18 g, and could easily be carried as a backpack by the pigeon, as shown in Figures 3C,D.

**Figure 4:**
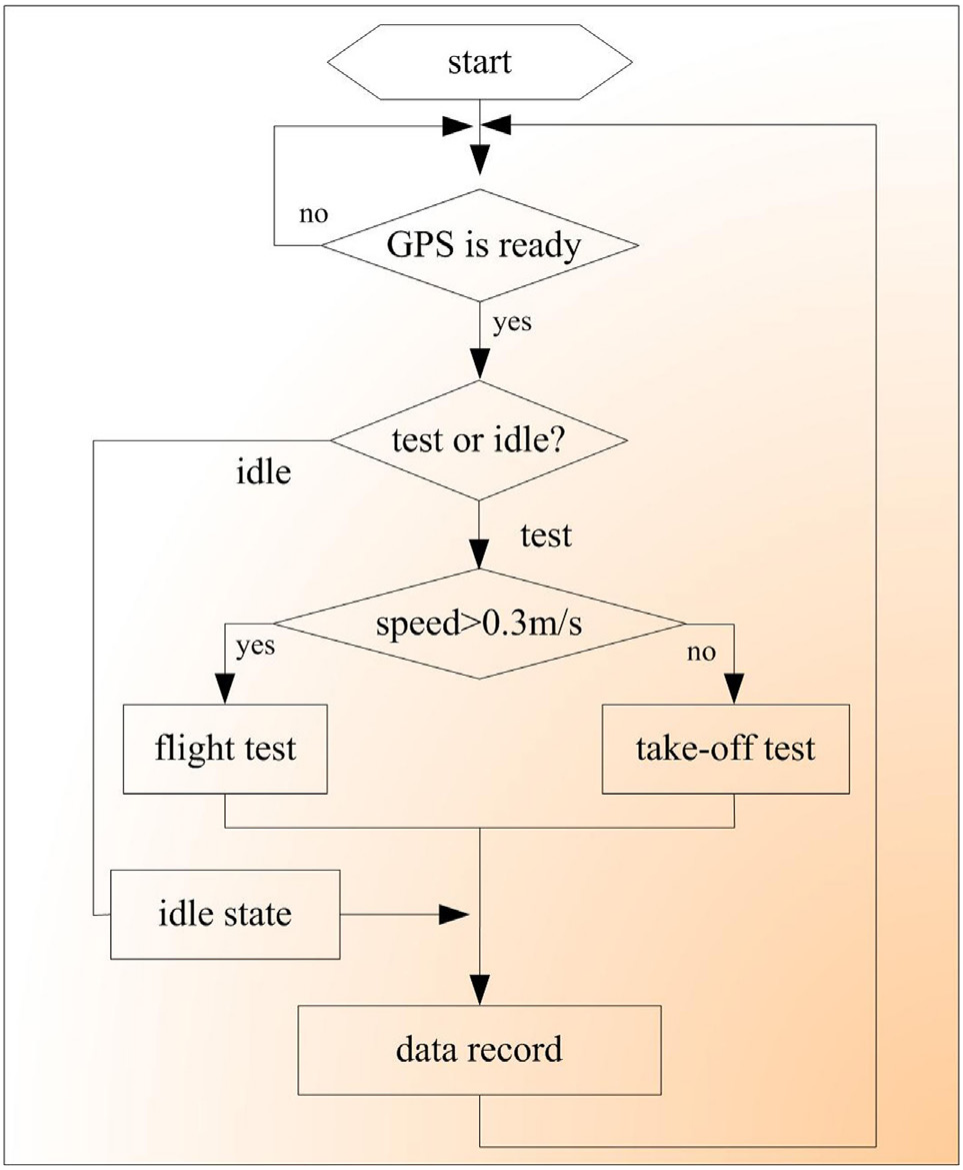
Flow diagram of robo-pigeon fight control in open space.

The microprocessor was preloaded with a C program to generate stimulation and record the data automatically following the process illustrated in Figure 4. As soon as a valid GPS position was received by the microprocessor through the serial port, it entered the test or idle state. The idle state was employed to avoid fatigue due to continuous testing. In the following test, the speed of the robo-pigeon was first determined based on the GPS data. If the speed was more than 0.3 m/s, the flight test was conducted as follows: stimulation was applied to the left or right DIVA nucleus according to the rules mentioned above. Otherwise, a take-off test involving PAG stimulation was conducted. In each test cycle, the GPS position data and corresponding stimulation parameters were arranged based on the format of Table 1 and written into a text file on the TF card. Only the GPS position data were recorded for the idle state.

**Figure 5:**
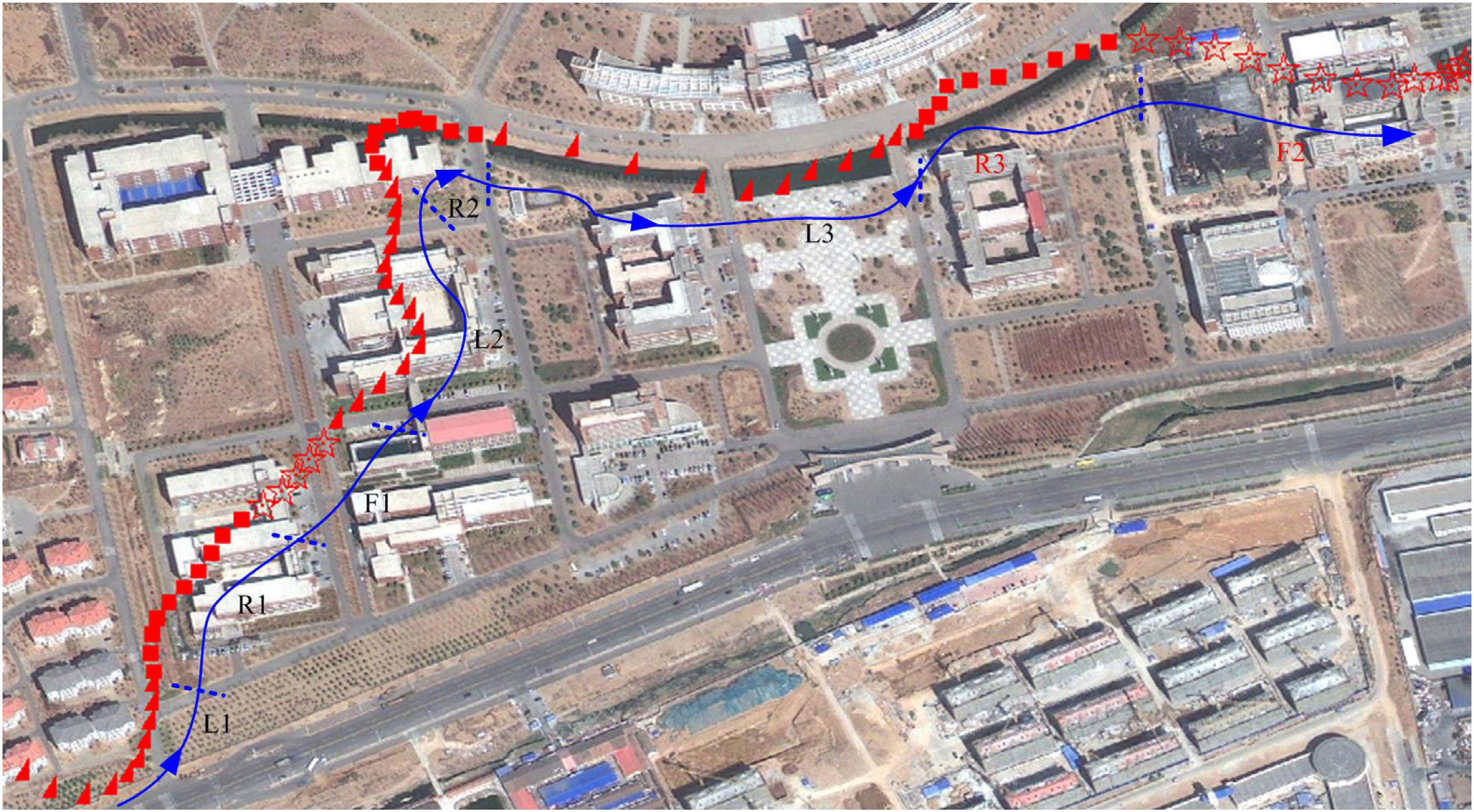
Results of robo-pigeon flight control test in open space.

When the test was completed, the text file on the TF card was transferred to a computer *via* a TF card reader and processed using a custom PC-based program to display the test results presented in Figure 5. The satellite picture was vectorized into a digital electronic map using SuperMap Deskpro 5.0 (SuperMap Software Co., Ltd.). Based on the data on the TF card, a series of vector graphs representing the flight control characteristics was constructed on the digital map using different types of identification dots. In Figure 5, each dot represents a position of the robo-pigeon during its flight, each ▲ denotes a position at which left DIVA stimulation was issued, each ■ represents a position at which right DIVA stimulation was issued, and each ☆ indicates a position at which no stimulation was issued.

For convenience of illustration, a solid auxiliary line with arrows was drawn manually and divided into several segments, which are labeled as L1, R1, F1, L2, R2, L3, R3, and F2 in Figure 5. Each segment corresponds to the command type and stimulation parameters listed in Table 2 according to the data recorded on the TF card.

**Table 2:** Effective stimulation parameters in the robo-pigeon flight control test.

Types	Command	Stimulation parameters		
Segment label	L/R/F/idle	Pulse number	Pulse duration	Pulse frequency
L1	L	20	4	100
R1	R	20	4	100
F1	Idle	–	–	–
L2	L	20	4	110
R2	R	20	4	110
L3	L	20	5	110
R3	R	20	5	110
F2	Idle	–	–	–

Command and stimulation are determined by a program running on the GPS stimulator, the whole vector graphs are drawn automatically by our custom PC-based program based on the GPS data on the TF card. Table 2 shows command and stimulation corresponding to flight trajectory in the Figure 5. L1 corresponds to left DIVA stimulation and contains ten points, each point represents a position on the digital map and has the same stimulation (pulse number = 20, pulse duration = 0.4 ms, pulse frequency = 100 Hz) shown in Table 2. R1 corresponds to right DIVA stimulation, it has the same relation between the stimulation and the GPS data with L1. F1 is corresponding to idle without stimulation. According to stimulation gradient rules mentioned above, the frequency was increased in increments of 10 and a new combination of parameters (pulse number = 20, pulse duration = 0.4 ms, pulse frequency = 110) was generated, L2 and R2 are flight trajectory corresponding to left and right DIVA stimulation with the new parameter. Then, the pulse duration was increased in increments of 1 (unit 0.1 ms). L3 and R3 are results of fight control with the new stimulation (pulse number = 20, pulse duration = 0.5 ms, pulse frequency = 110).

According to the above results, the following conclusions can be drawn: (1) the tested robo-pigeon can be controlled in left and right direction in open space, (2) the multiple parameter combinations shown in Table 2 are effective for the tested robo-pigeon, (3) the minimal stimulus parameters (pulse number = 20, pulse duration = 0.4 ms, pulse frequency = 100 Hz) could be determined as the optimized stimulus parameters for flight control of the robo-pigeon, taking into account energy consumption and potential nerve damage.

**Figure 6:**
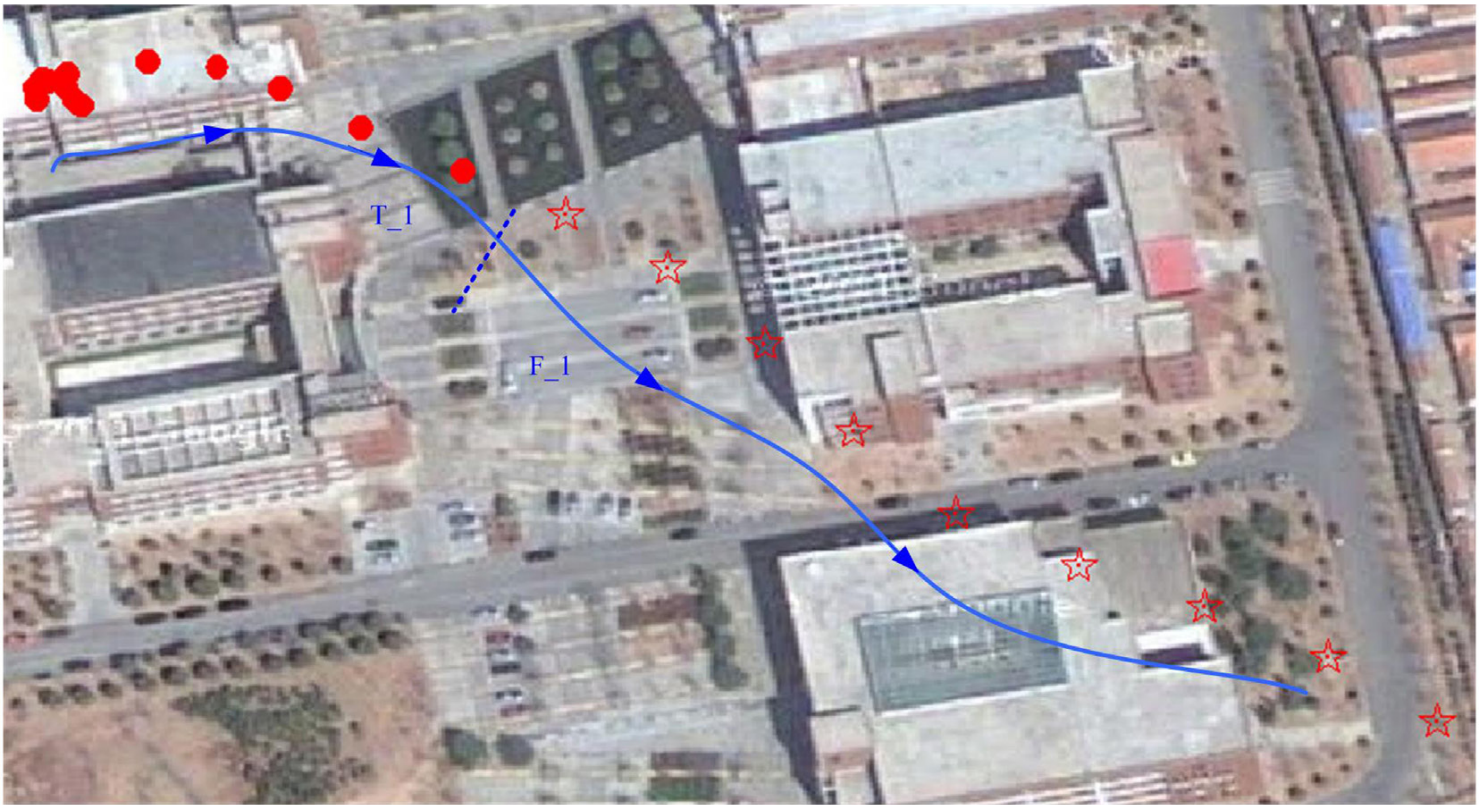
Results of robo-pigeon take-off test in open space.

A solid auxiliary line with arrows was drawn manually and divided into two segments, which are labeled as T_1 and F_1 in Figure 6. T_1 demonstrates an effective take-off using the stimulation listed in Table 3 according to the data recorded on the TF card. F_1 is corresponding to idle without stimulation. The stimulus parameters (pulse number = 20, pulse duration = 0.6 ms, pulse frequency = 110 Hz) could be determined as the optimized stimulus parameters for take-off of the robo-pigeon.

**Table 3:** Effective stimulation parameters in the robo-pigeon take-off test.

Types	Command	Stimulation parameters		
Segment label	T/idle	Pulse number	Pulse duration	Pulse frequency
T_1	T	20	6	110
F_1	Idle	–	–	–

Results such as those obtained in this study will enable researchers to analyze and evaluate the control characteristics of robo-pigeons and optimize the parameter settings for each stimulation channel. Furthermore, such results will facilitate the identification of problems that could not be exposed in laboratory environments and the development of strategies to improve automatic robo-pigeon navigation in potential applications.

## Discussion

The stimulus parameters and position are not recorded synchronously due to difficulties of positioning a flying robo-pigeon in the indoor environment, so, the results of flight control experiment in the laboratory need to be manually analyzed and judged by the experimenter. Moreover, it is impossible to draw accurately the motion trajectory of robo-pigeons only based on experimental videos, in addition, this work is time consuming and laborious. Therefore, the experimental results cannot be objectively displayed and quantitatively analyzed. More unfortunately, those experimental data are not complete due to space constraints in the laboratory. So, it does not apply to open space, which is actual environment of potential application of robo-pigeons. In view of the above deficiencies, we proposed a method based on the newly designed GPS-based stimulator, it has the capabilities of collecting experimental data and generating visual trajectory of robo-pigeons automatically.

Random errors originating from the electrode positioning, electrode coating, and devices used for robo-pigeon development are inevitable. Those errors cause the parameter settings to differ between individuals and could even lead to the functional failure of a robo-pigeon. Therefore, it is essential to test and evaluate the control characteristics of robo-pigeons in open space. The method proposed just provides a simple and feasible solution to the complex and tedious tests.

The system described in this report overcomes some of the drawbacks of previous telemetry systems by providing an evaluation method of robo-pigeon flight control testing in open space. A special feature of the system is that it can record experimental data and display experimental results automatically. The system is not limited by the experimental environment and will enable researchers to evaluate the control characteristics of robo-pigeons in open space quickly and clearly, to optimize the parameter settings for every stimulation channel, and to identify methods of improving the robo-pigeon design process. Simultaneously, the system will enrich brain stimulation research methods and enable bio-robot experiments to be conducted in open environments.

## Ethics Statement

Experiments were done on adult pigeons, microelectrodes were implanted into three emotion-related nuclei in pigeon’s brain: left and right DIVA and PAG. This study was approved by the Ethics Committee of Shandong University of Science and Technology (Agreement number: SDUST201406-1-036). All of the experiments were performed in accordance with the guidelines issued by the Ethics Committee of Shandong University of Science and Technology, and they complied with the Guidelines for the Use of Animals of the International Brain Research Organization. All of the pigeons used in this study were well cared for and lived in groups in a special dovecote, never sacrificed.

## Author Contributions

Conception or design of the work: JY, WL, RH, ZW, and XS. Experiment: JY, WL, RH, HW, MS, and XS. Data analysis: JY, WL, HW, XS, and MS. Drafting the article: JY, RH, HW, WL, and MS. Critical revision of the article: WL, JY, ZW, and XS. All the authors have approved the final manuscript.

## Conflict of Interest Statement

The authors declare that the research was conducted in the absence of any commercial or financial relationships that could be construed as a potential conflict of interest.
